# SG formation relies on eIF4GI-G3BP interaction which is targeted by picornavirus stress antagonists

**DOI:** 10.1038/s41421-018-0068-4

**Published:** 2019-01-01

**Authors:** Xiaodan Yang, Zhulong Hu, Qiang Zhang, Shanshan Fan, Yi Zhong, Dong Guo, Yali Qin, Mingzhou Chen

**Affiliations:** State Key Laboratory of Virology and Modern Virology Research Center, College of Life Sciences, Wuhan University, LuoJia Hill, Wuhan 430072 China

**Keywords:** Stress signalling, Ribosome

## Abstract

Typical stress granules (tSGs) are stalled translation pre-initiation complex aggregations in the cytoplasm, and their formation is a common consequence of translation initiation inhibition under stress. We previously found that 2A protease of picornaviruses blocks tSG formation and induces atypical SG formation, but the molecular mechanism by which 2A inhibits tSG formation remains unclear. Here, we found that eukaryotic translation initiation factor 4 gamma1 (eIF4GI) is critical for tSG formation by interacting with Ras-GTPase-activating protein SH3-domain-binding protein (G3BP), and this interaction is mediated by aa 182–203 of eIF4GI and the RNA-binding domain of G3BP. Upon eIF4GI-G3BP interaction, eIF4GI can assemble into tSGs and rescue tSG formation. Finally, we found that 2A or L protein of picornaviruses blocks tSG formation by disrupting eIF4GI-G3BP interaction. Our findings provide the first evidence that eIF4GI-G3BP interaction is indispensable for tSG formation, and 2A or L protein of picornaviruses interferes eIF4GI-G3BP interaction, thereby blocking tSG formation.

## Introduction

Stress granules (SGs) are non-membranous, transiently assembled cytoplasmic aggregates, which consist of stalled translation preinitiation complexes (PICs) and where modulate cell signaling by sequestering key signal transduction proteins. SGs are also considered to be the sites for mRNA storage and sorting, which results in a rapid re-initiation of translationally arrested mRNAs once the stress has been resolved^1^. The oligomerization of several SG-nucleating factors, such as Ras GTPase-activating protein-binding protein (G3BP) and T-cell intracellular antigen (TIA-1), is required for SG formation^2–4^. However, some SG components and markers may be passively recruited to SGs as a consequence of mRNA accumulation at these foci. For example, knockdown or overexpression of poly(A)-binding protein 1 (PABP1) and/or PABP4 has no effect on SG formation or mRNA localization to SGs^5^, suggesting that PABPs are not required for SG formation.

Host translational machinery is indispensable for the whole replication cycle of viruses, and SG plays a critical role in host antiviral defense. To antagonize host defense, many viruses have hence evolved various strategies to disrupt SG formation for releasing transcripts from being stalled in SGs and for efficient translation of their proteins^6,7^. For example, nonstructural protein 3 of the Old World alphaviruses Semliki Forest virus and Chikungunya virus sequester G3BP by forming a complex and inhibit SG formation induced by both viral infection and other stresses^8,9^. West Nile virus and dengue virus impede SG formation by sequestering TIA-1 through specific interaction with the ends of viral minus-strand RNAs^10^. Gag protein of human immunodeficiency virus 1 prevents SG formation by interacting with and recruiting Staufen1 to ribonucleoproteins for encapsidation of viral genomic RNA^11^. Core protein of Japanese encephalitis virus inhibits SG formation by directly interacting with Caprin1, a component of SGs^12^. Inclusion bodies of human parainfluenza virus type 3 inhibit antiviral SG formation by shielding viral RNAs^13^.

Enterovirus 71 (EV71) belongs to the genus Enterovirus in the family of Picornaviridae, which also includes coxsackievirus, poliovirus (PV), and human rhinovirus. EV71 can cause hand-foot-mouth disease in infants and sometimes more severe neurological disease that can result in death^14^. Previous studies showed that picornaviruses block typical SG (tSG) formation via cleavage of G3BP by 3C protease^15–17^. But other picornaviruses such as Theiler’s murine encephalomyelitis virus and encephalomyocarditis virus, inhibit tSG formation by L protein without cleaving G3BP^18,19^. Furthermore, we demonstrated that 2A, but not 3C, blocked tSG formation and induced atypical stress granule (aSG) formation in EV71-infected cells, and aSGs are different from tSGs in that aSG formation is independent of eIF2α phosphorylation and aSG cannot be disassembled by cycloheximide^20^. Infection of 2A protease activity-inactivated recombinant EV71 (EV71-2A^C110S^) induced tSG formation but failed to induce aSG formation. Furthermore, the 2A protease of other picornaviruses such as PV and CVA also induced aSG formation and blocked tSG formation. We found that cleavage of eukaryotic translation initiation factor 4 gamma 1 (eIF4GI) by 2A of picornaviruses is critical for the induction of aSG formation. Expression of eIF4GI^G689E^ eliminated the induction of aSG formation by 2A, but was unable to recover tSG formation in the presence of sodium arsenite (AS)^20^; thus, the inhibitory effect of 2A on tSG formation is complicated. In this study, we confirmed that eIF4GI-G3BP interaction is critical for the formation of tSGs under environmental stress, and disruption of eIF4GI-G3BP interaction by 2A can block tSG formation.

## Results

### eIF4GI is required for tSG formation

**Fig. 1:**
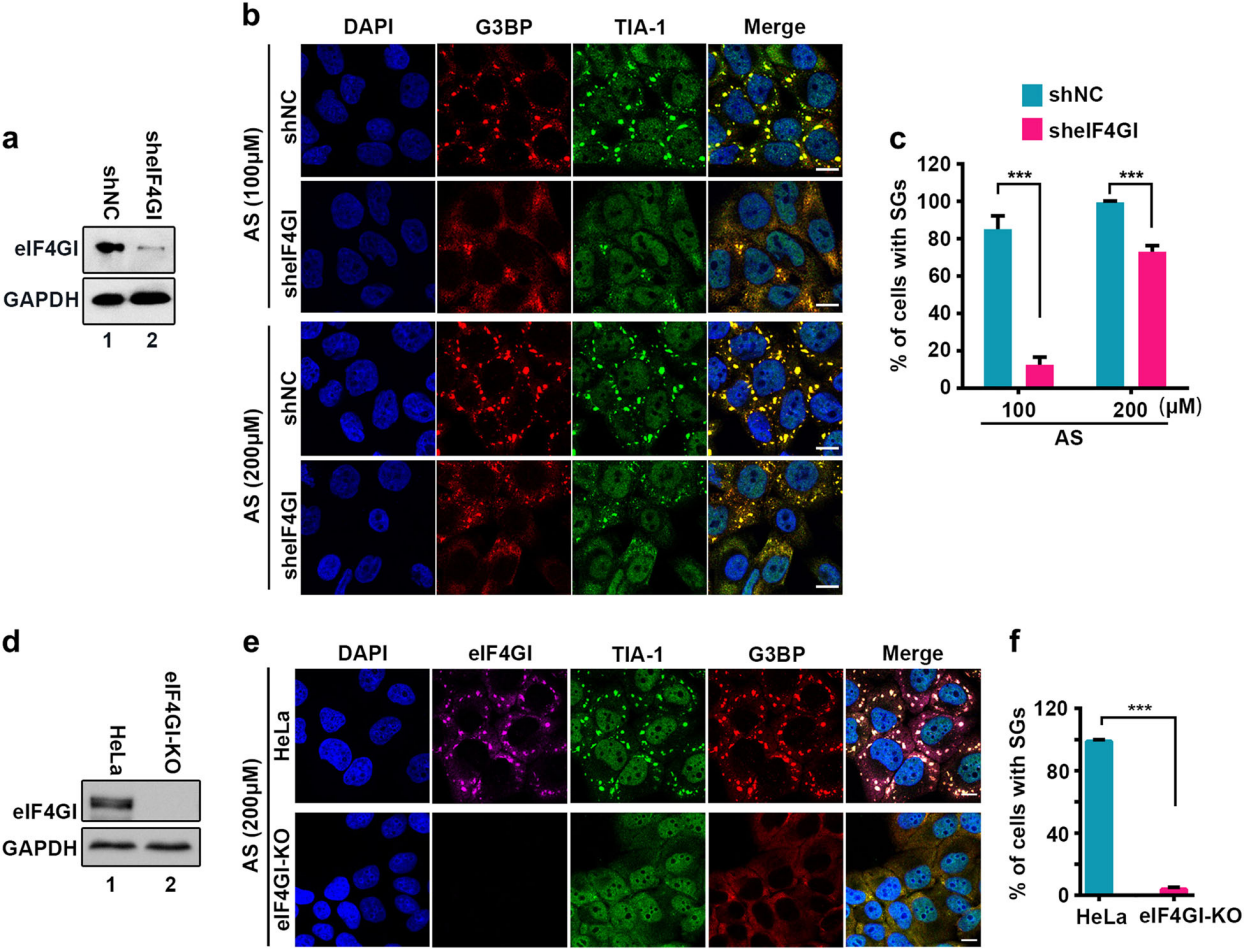
eIF4GI is indispensable for the formation of tSGs. **a**–**c** Effects of eIF4GI KD on tSG formation. Examination of the protein level of eIF4GI in HeLa cells with KD of eIF4GI (**a**). Cells were treated with AS for 1 h, then fixed and stained with G3BP (red) and TIA-1 (green). IF (**b**) and quantitation (**c**) analysis of cells with tSGs (G3BP served as a marker). *n* = 3, 300 cells/assay were counted, mean ± SD; ***p < 0.001. **d**–**f** Effects of eIF4GI KO on tSG formation. Examination of the protein level of eIF4GI in HeLa cells with KO of eIF4GI (HeLa-eIF4GI-KO cells) (**d**). Cells were treated with AS for 1 h and then fixed and stained with eIF4GI (purple), G3BP (red), and TIA-1 (green). IF (**e**) and quantitation (**f**) analysis of cells with tSGs (G3BP served as a marker). *n* = 3, 300 cells/assay were counted, mean ± SD; ***p < 0.001. Scale bars, 10 μm. See also Supplementary Fig. S1

Our previous study showed that 2A protease activity of EV71 is essential for the blockage of tSG formation^20^. Thus, we sought to determine the molecular mechanism by which 2A blocks tSG formation. We suspected that 2A cleaves or disrupts the function of the critical tSG formation factor(s), resulting in the blockage of tSG formation. To test this hypothesis, we knocked down (KD) nine previously reported substrates of 2A^21–29^ and found that AS-induced tSG formation was sharply weakened upon KD of eIF4GI, but KD of eIF4GII, PAPB, NUP62, NUP98, SQSTM1, FBP1, GAB1, or SRF had no effect on AS-induced tSG formation (Fig. 1a–c and Supplementary Fig. S1a), suggesting that eIF4GI is a critical factor for tSG formation. To validate the critical role of eIF4GI in the formation of tSG, we used the CRISPR/Cas9 system to construct eIF4GI knockout (KO) HeLa cells (HeLa-eIF4GI-KO) and found that AS-induced tSG formation was abolished in HeLa-eIF4GI-KO cells (Fig. 1d–f). Furthermore, in eIF4GI-KO/non-KO co-cultured cells, AS induced tSG formation in non-KO cells, but not in eIF4GI-KO cells (Supplementary Fig. S1b). Taken together, these results demonstrate that eIF4GI is indeed required for tSG formation.

### eIF4GI interacts with G3BP

**Fig. 2:**
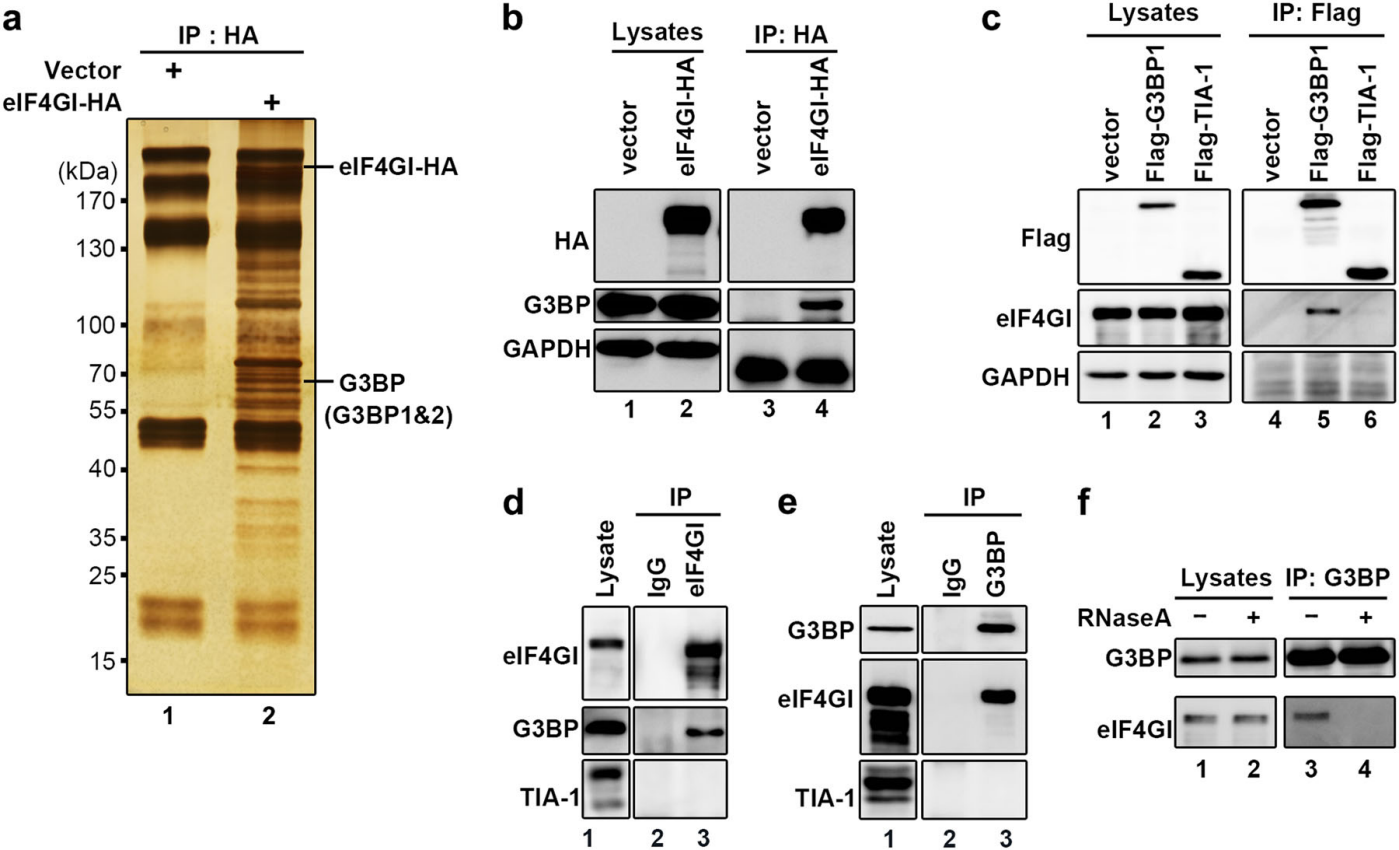
eIF4GI interacts with G3BP. **a**–**b** HeLa cells were transfected with eIF4GI-HA for 24 h, and cell lysates were subjected to IP/MS with anti-HA antibody. Immunoprecipitates were separated via SDS-PAGE and analyzed via silver staining (**a**) or WB (**b**).The anti-G3BP antibody, which used to detect endogenous G3BP, can recognize both G3BP1 and G3BP2. **c** To detect the interaction of G3BP or TIA-1 with endogenous eIF4GI, HeLa cells expressing Flag-tagged G3BP1 or TIA-1 were lysed and subjected to IP with anti-Flag antibody, followed by WB to resolve the immune complexes. **d**–**e** Interaction examination between endogenous eIF4GI and G3BP (TIA-1 was used as negative control). HeLa cells were lysed and subjected to IP with antibodies against eIF4GI (**d**) or G3BP (**e**). Lysates and immunoprecipitates were resolved via WB with indicated antibodies. **f** HeLa cells were lysed and treated with RNaseA ( + ) or mock-treated (−) before IP with anti-G3BP antibody, followed by detection of eIF4GI and G3BP via Western blots. See also Supplementary Fig. S2

Next, we sought to determine the role of eIF4GI in tSG formation. Because tSG formation is regulated by protein–protein interactions between SG formation components, we suspected that eIF4GI regulates tSG formation by its interaction with other factor(s) that are critical for SG formation. We over-expressed HA-tagged eIF4GI as a bait protein and then performed immunoprecipitation (IP) using an antibody against HA, followed by mass spectrometry, and found that eIF4GI interacted with both G3BP1 and G3BP2, which are critical tSG formation factors (Fig. 2a, b). To confirm the specificity of eIF4GI-G3BP interaction, we also expressed Flag-tagged G3BP1 (as a representative of G3BP) or TIA-1 (TIA-1 is also a critical tSG formation factor) for co-IP assays. The results showed that Flag-G3BP1, but not Flag-TIA-1, interacted with endogenous eIF4GI (Fig. 2c). Furthermore, endogenous eIF4GI also interacted with G3BP, but not with TIA-1 (Fig. 2d, e). Furthermore, we found that eIF4GI-G3BP interaction is RNA-dependent since treatment with RNase A abolished eIF4GI-G3BP interaction (Fig. 2f). Similar results were also obtained in RD cells (Supplementary Fig. S2). Taken together, these results show that eIF4GI interacts with G3BP, but not TIA-1, in an RNA-dependent manner.

### eIF4GI-G3BP interaction is indispensable for tSG formation

Previous studies reported that G3BP is critical for tSG formation^3,30^; however, the molecular mechanism by which G3BP regulates tSG formation has not been fully elucidated. Thus, we explored the possibility that eIF4GI-G3BP interaction contributes to tSG formation.

**Fig. 3:**
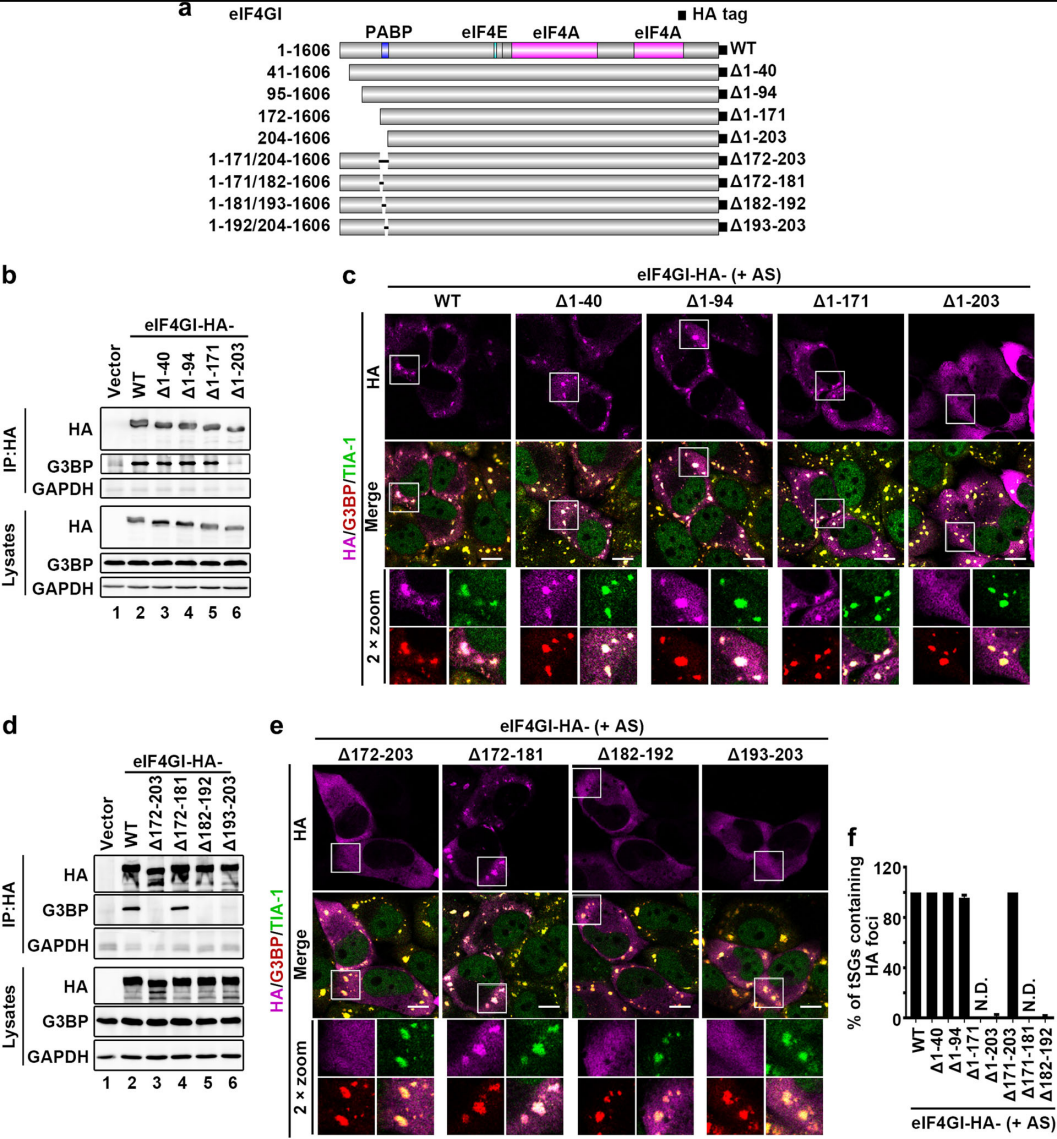
eIF4GI-G3BP interaction is required for eIF4GI assembly into tSGs. **a** Graphic description of eIF4GI and its mutants. **b**–**f** eIF4GI-HA and its mutants were expressed as indicated. Cell lysates were subjected to IP and analyzed as in Fig. 2b (**b** and **d**). Cells were treated with 200 μM AS for 1 h, then fixed and stained with HA (purple), G3BP (red), and TIA-1 (green). G3BP and TIA-1 served as indicators of tSGs (**c** and **e**). Quantitation of SGs containing indicated HA foci in **c** and **e** (**f)**. *n* = 3, 300 cells/assay were counted, mean ± SD, N.D., not detectable. Scale bars, 10 μm. Supplementary Fig. S3

**Fig. 4:**
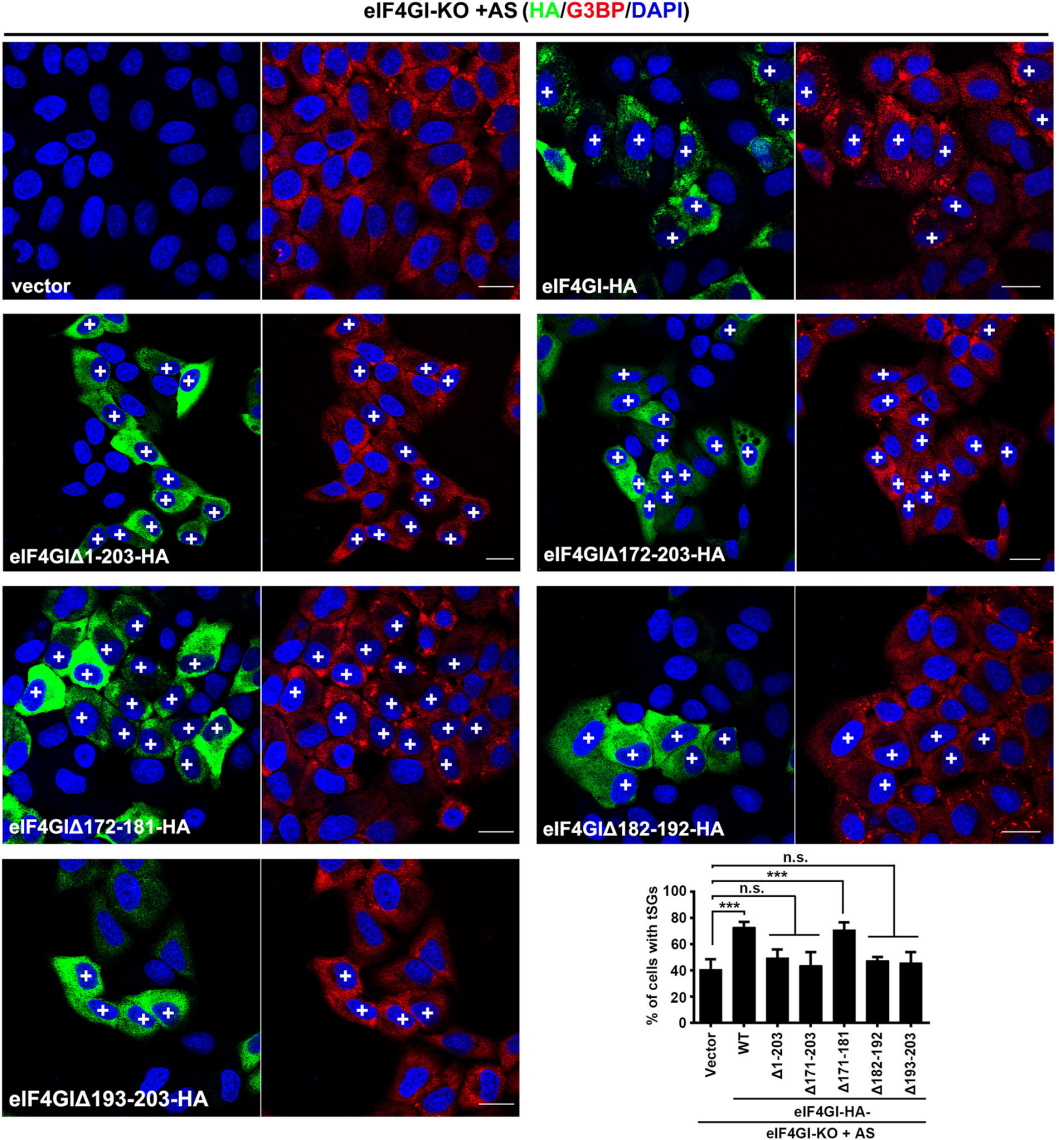
tSG formation rescue assays in HeLa-eIF4GI-KO cells. HeLa-eIF4GI-KO cells were transfected with HA-tagged eIF4GI or its mutants as indicated for 24 h, treated with AS for 1 h, and then subjected to IF assay. Cells were stained with HA (green), G3BP (red), and DAPI (blue). “ + ” indicates cells expressing HA-tagged protein. Quantitation of HA-positive cells with G3BP-marked tSGs (bottom and right panel). For “Vector” column, the overall percentage of cells with SGs was calculated. *n* = 3, 400 cells/condition were counted, mean ± SD; ***p < 0.001, *n.s.* no statistical significance. Scale bars, 10 μm

First, we identified the critical region within eIF4GI for regulating eIF4GI-G3BP interaction and tSG formation. In *homo sapiens*, eIF4GI has eight transcription variants resulting in translation products with five different N-termini^31^. Thus, in addition to HA-eIF4GI (1–1606), we constructed four other HA-tagged eIF4GI variants with different N-termini (eIF4GIΔ1-40, eIF4GIΔ1-94, eIF4GIΔ1-171, and eIF4GIΔ1-203) (Fig. 3a) and assessed their ability to bind to G3BP, and found that only the eIF4GIΔ1-203 variant failed to interact with G3BP (Fig. 3b). Correspondingly, when HeLa cells expressing each variant of eIF4GI were treated with AS, all the eIF4GI variants that interacted with G3BP were assembled into AS-induced tSGs, but eIF4GIΔN203 remained diffuse and failed to localize to tSGs (Fig. 3c and f). More precise mapping showed that neither eIF4GIΔ182-192 nor eIF4GIΔ193-203 interacted with G3BP and localized to tSGs (Fig. 3d–f), suggesting that amino acids (aa) 182–203 within eIF4GI are indispensable for eIF4GI interaction with G3BP and assembly into tSGs. To confirm that aa 182–203 of eIF4GI are critical for tSG formation, we performed tSG formation function rescue assays by expressing eIF4GI variants or mutants in HeLa-eIF4GI-KO cells in the presence of AS. We found that as long as eIF4GI variants or mutants lost the G3BP interaction region, they failed to rescue tSG formation (Fig. 4). Taken together, these data demonstrate that eIF4GI is critical for tSG formation via eIF4GI-G3BP interaction. Since the G3BP interaction domain is very close to the PABP interaction domain^32^, it raises the question whether eIF4GI-G3BP interaction is mediated by PABP. Therefore, we depleted PABP via shRNA and found that eIF4GI-G3BP interaction was not inhibited (Supplementary Fig. S3), indicating that eIF4GI-G3BP interaction is not mediated by PABP.

**Fig. 5:**
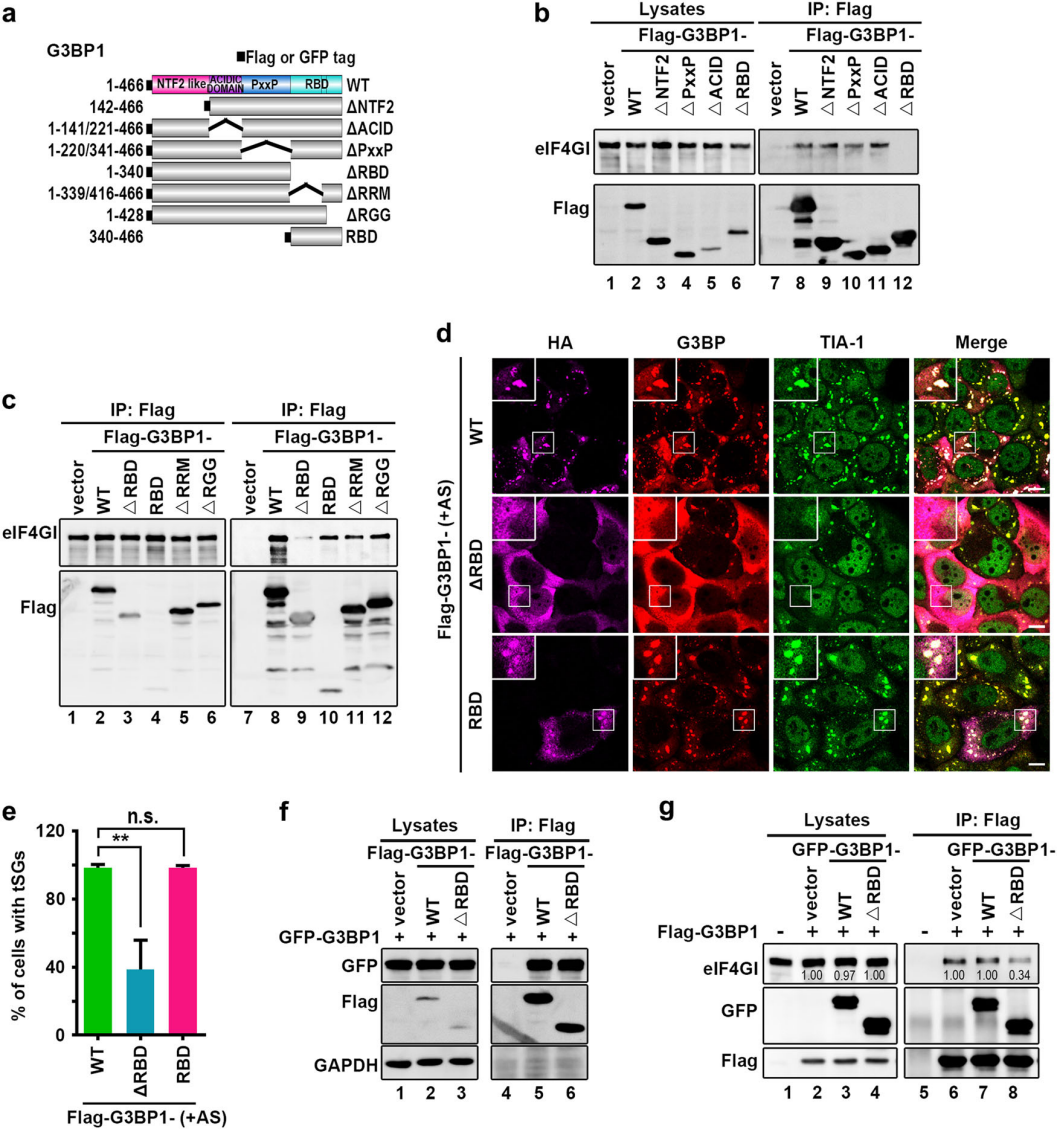
RBD of G3BP is required for eIF4GI-G3BP interaction. **a** Graphic description of G3BP and its mutants. **b** and **c** HeLa cells were transfected as indicated, and cell lysates were processed as in Fig. 2c. **d**–**e** HeLa cells were transfected as indicated, treated with AS (200 μM, 1 h), and stained with TIA-1 (green) and G3BP (red) to visualize the tSGs and flag (purple) to visualize the expression of G3BP and its mutants (**d**). Quantitation of Flag-positive cells with TIA-1-marked tSGs (**e**). *n* = 3, 240 cells/condition were counted, mean ± SD; ***p < 0.001, *n.s.* no statistical significance. **f**–**g** HeLa cells transfected as indicated, and cell lysates were processed as in (**b**). Scale bars, 10 μm

Second, we identified the critical region within G3BP for regulating eIF4GI-G3BP interaction and tSG formation. We also take G3BP1 as a representative of G3BP. Flag-tagged G3BP1 lacking the nuclear transport factor 2 domain, acidic (ACID) domain, proline-rich (PxxP) domain, or RNA-binding domain (RBD) were constructed and expressed (Fig. 5a)^3^, and only G3BP1-ΔRBD lost the eIF4GI-binding activity (Fig. 5b). More precise mapping showed that RBD alone, G3BP1 lacking the RNA recognition motif (G3BP1-ΔRRM) and G3BP1 lacking the Arg-Gly-Gly-rich motif (G3BP1-ΔRGG) were still able to interact with eIF4GI (Fig. 5c), indicating that the whole RBD is required and sufficient to mediate eIF4GI-G3BP1 interaction. Furthermore, we evaluated the effect of RBD-mediated eIF4GI-G3BP1 interaction on tSG formation. HeLa cells expressing G3BP1-ΔRBD or G3BP1-RBD were treated with AS, and we found that G3BP1-ΔRBD, but not G3BP1-RBD, inhibited tSG formation (Fig. 5d, e), suggesting that, instead of binding to eIF4GI directly, G3BP1-ΔRBD disrupts the eIF4GI-G3BP interaction to inhibit tSG formation by other means, such as interrupting the function of G3BP. Subsequently, G3BP1-ΔRBD was able to bind to G3BP1 normally (Fig. 5f) and inhibited eIF4GI-G3BP interaction (Fig. 5g). Taken together, these results demonstrate that G3BP1 interacts with eIF4GI via the RBD, and G3BP1-ΔRBD binding to G3BP1 disrupts eIF4GI-G3BP interaction, resulting in the blockage of tSG formation.

### 2A or L protein of picornaviruses blocks SG formation by disrupting eIF4GI-G3BP interaction

**Fig. 6:**
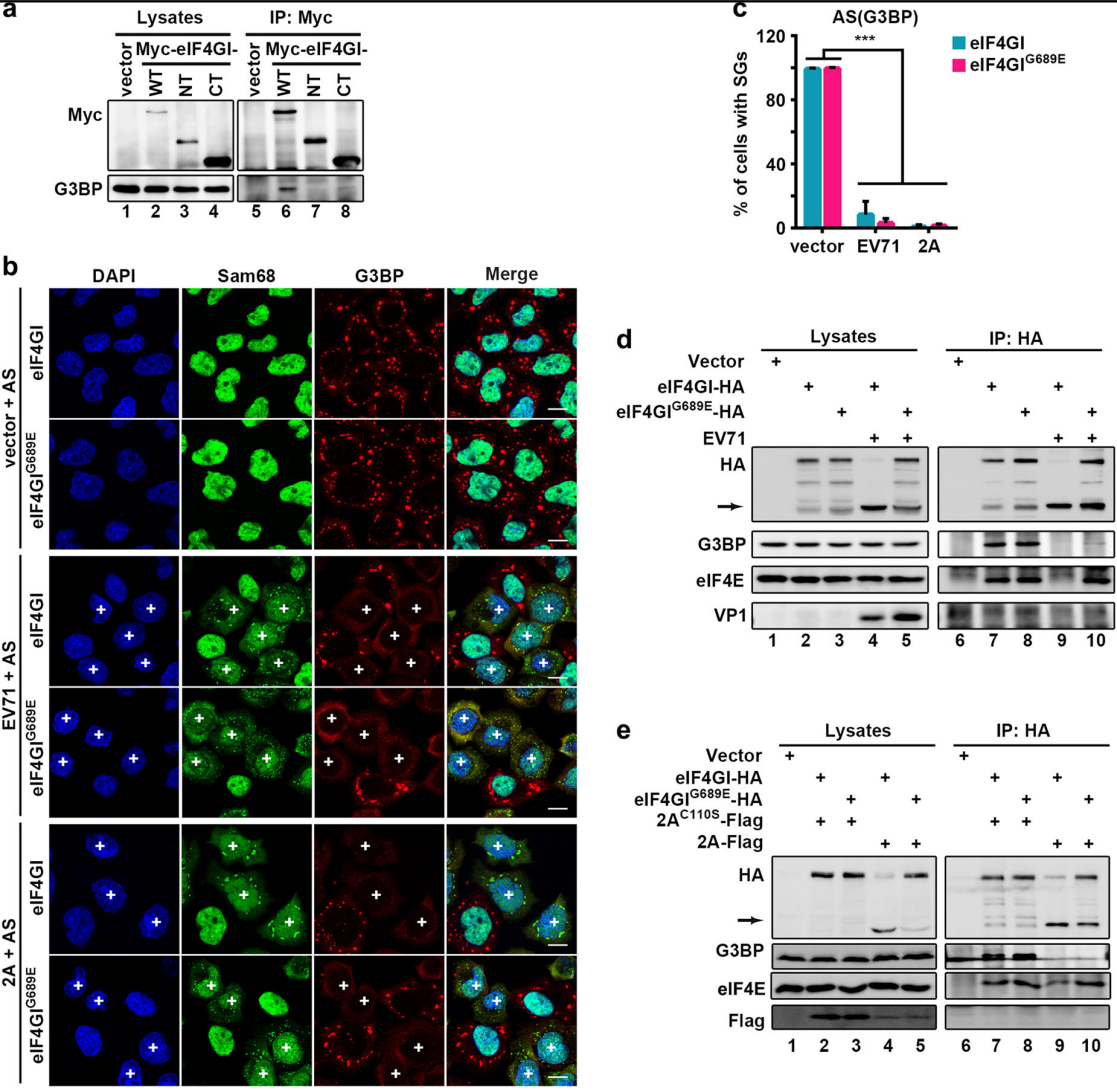
EV71 and 2A block tSG formation by disrupting eIF4GI-G3BP interaction. **a** HeLa cells were transfected as indicated. Cell lysates were subjected to IP with an anti-Myc antibody. **b** eIF4GI-HA- or eIF4GI^G689E^-HA-HeLa cells were transfected with vector or 2A for 24h, or infected with EV71 (MOI = 10) for 5 h, then treated with 200 μM AS for 1 h, and stained with Sam68 (green) and G3BP (red). Sam68 served as an indicator of EV71 infection or 2A expression. “ + ” indicates EV71-infected or 2A-expressing cells. **c** Quantitation analysis of vector-transfected, EV71-infected, or 2A-expressing cells with tSGs in (**b**). *n* = 3, 240 cells/condition were counted, mean ± SD; ***p < 0.001. **d** eIF4GI-HA- or eIF4GI^G689E^-HA-HeLa cells were infected with EV71 as indicated, and cell lysates were subjected to IP with an anti-HA antibody. The bound proteins were analyzed via WB. VP1 indicated EV71 infection. Arrow indicates eIF4GI cleavage products. **e** eIF4GI-HA- or eIF4GI^G689E^-HA-HeLa cells were transfected with Flag-tagged 2A or 2A^C110S^ for 24 h and analyzed as in (**d**). Scale bars, 10 μm

Having found that eIF4GI-G3BP interaction is critical for tSG formation, we sought to determine whether 2A of EV71 blocks tSG formation by disrupting eIF4GI-G3BP interaction. Because 2A of EV71 cleaves eIF4GI into two fragments, N-terminus (eIF4GI-NT) and C-terminus (eIF4GI-CT), we over-expressed eIF4GI, eIF4GI-NT, or eIF4GI-CT and found that eIF4GI, but not eIF4GI-NT or eIF4GI-CT, interacted with G3BP (Fig. 6a), suggesting that full-length eIF4GI is required for eIF4GI-G3BP interaction, and 2A cleavage of eIF4GI abolishes eIF4GI-G3BP interaction.

**Fig. 7:**
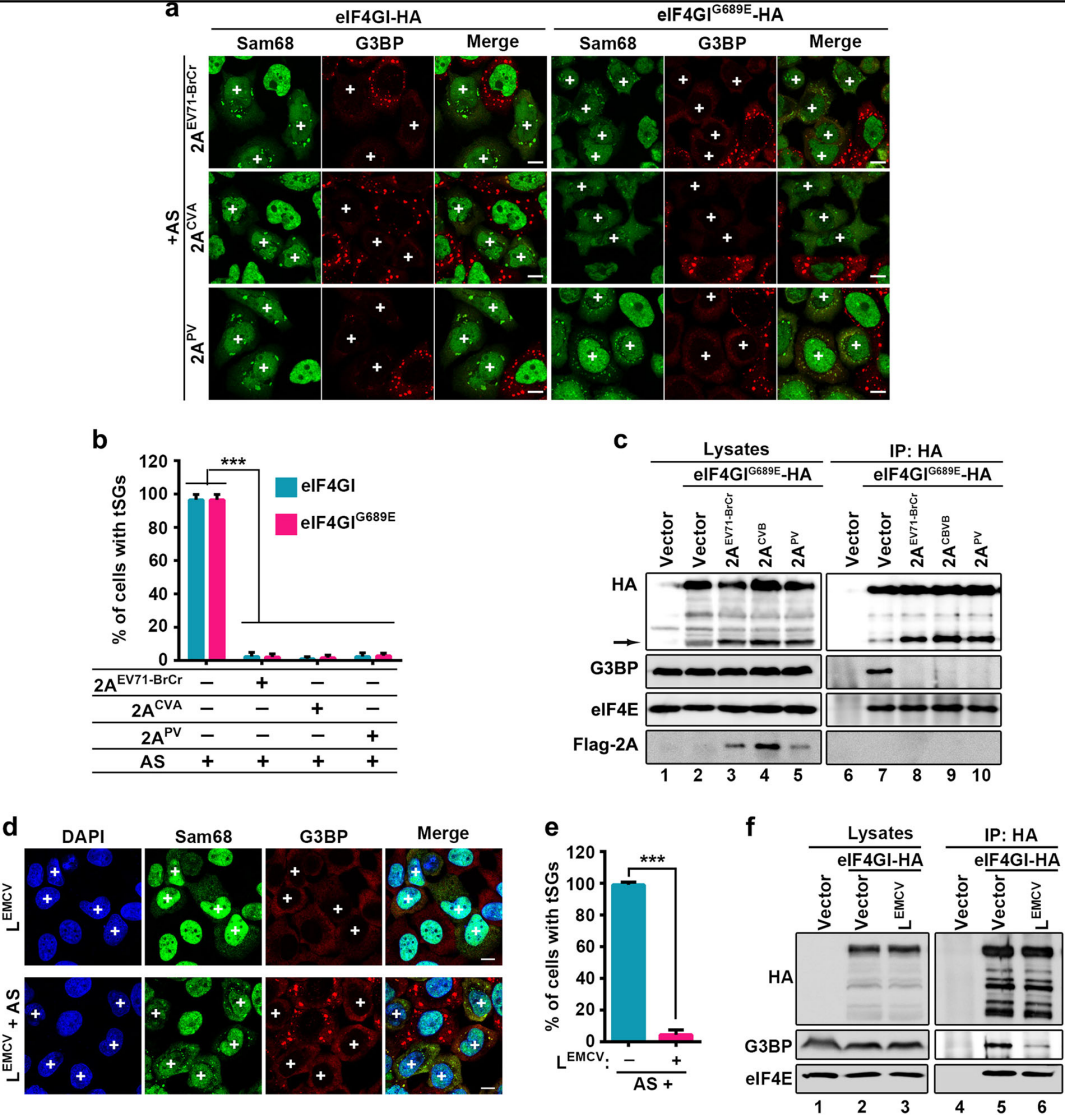
Disruption of eIF4GI-G3BP interaction by 2A/L to block tSG formation is common among picornaviruses. **a** and **b** eIF4GI-HA- and eIF4GI^G689E^-HA-HeLa cells were transfected with 2A of EV71-BrCr, CVA, or PV for 24 h, and cells were processed as in Fig. 6a (**a**) and quantified as in Fig. 6b (**b**). (**c**) eIF4GI^G689E^-HA-HeLa cells were transfected with 2A of EV71-BrCr, CVA, or PV for 24 h and analyzed as in Fig. 6d. (**d**) HeLa cells were transfected with L^EMCV^ for 24 h, then treated with 200 μM AS or not. Cells were stained with Sam68 (green) to visualize expression of L^EMCV^ and G3BP (red) to visualize tSGs. **e** Quantitation of L^EMCV^-expressing cells with tSGs in (**d**). *n* = 3, 240 cells/condition were counted, mean ± SD; ***p < 0.001. **f** eIF4GI-HA-HeLa cells were transfected with L^EMCV^ for 24 h, and cell lysates were subjected to IP as in (**b**). “ + ” indicates 2A or L^EMCV^-expressing cells. Scale bars, 10 μm

2A protease cleaves eIF4GI at the unique site between R688 and G689, but produces multiple cleavage products of eIF4GI^33^, which is because of the cleavage of different eIF4GI isoforms at the same site. We generated the 2A cleavage-resistant mutation of eIF4GI, eIF4GI^G689E^, based on previous study^23^ and have also compared the cleaving efficiency of wild-type eIF4GI and G689E mutant by 2A and found that eIF4GI^G689E^ was indeed resistant to 2A cleavage^20^. We previously showed that 2A still inhibited tSG formation in spite of eIF4GI^G689E^ expression^20,23^. To confirm whether 2A inhibits tSG formation upon expression of eIF4GI^G689E^, we performed a similar assay using EV71-infected or 2A-transfected cells stably expressing eIF4GI^G689E^. Localization and punctate aggregation of Sam68 from the nucleus to cytoplasm was indicated as EV71-infected or 2A-expressing cells. Indeed, we also found that tSG formation was inhibited upon EV71 infection or 2A expression (Fig. 6b, c). To elucidate why 2A expression or EV71 infection can disrupt the tSG formation function of eIF4GI^G689E^, we performed co-IP assays using anti-HA antibody in HeLa cells stable expressing eIF4GI or eIF4GI^G689E^ with a C-terminal HA-tag. Previous studies showed that eIF4E interacts with N-terminus of eIF4GI^34,35^, as a result, only full-length eIF4GI-HA could interact with eIF4E. Thus, we used eIF4E as a positive control to detect protein interaction with full-length eIF4GI. We found that eIF4GI and eIF4GI^G689E^ interacted with G3BP and eIF4E (Fig. 6d, lanes 7 & 8). But in EV71-infected or 2A-expressing cells, eIF4GI-HA failed to co-IP G3BP or eIF4E due to 2A cleavage of eIF4GI; surprisingly, although eIF4GI^G689E^ is resistant to cleavage by 2A, it no longer interacted with G3BP, while still interacted with eIF4E (Fig. 6d, e, lanes 9 & 10), suggesting that 2A blocks eIF4GI/eIF4GI^G689E^-G3BP interaction. As a control, 2A^C110S^ could not block tSG formation and had no effect on eIF4GI/eIF4GI^G689E^-G3BP interaction (Fig. 6e, lanes 7 & 8). Furthermore, we previously found that 2A of EV71-BrCr, PV, and CVA were all capable of cleaving eIF4GI and blocking tSG formation^20^. Correspondingly, 2A of all these picornaviruses could still block tSG formation and disrupt eIF4GI^G689E^-G3BP interaction in HeLa-eIF4GI^G689E^ cells (Fig. 7a–c).

**Fig. 8:**
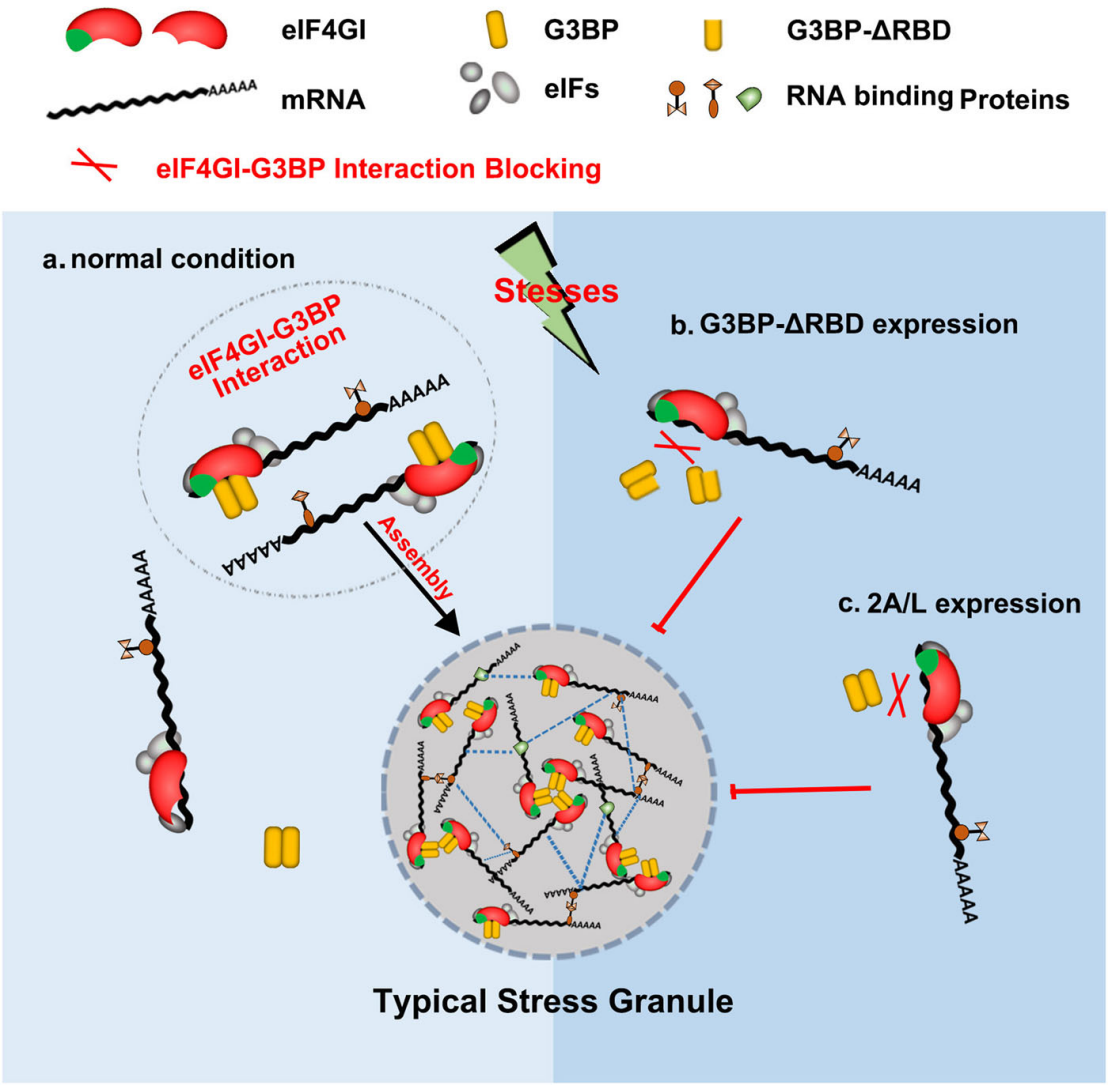
Models of the regulation of tSG assembly by eIF4GI-G3BP interaction. **a** In normal condition, stresses promote the interaction between eIF4GI and G3BP, which leads to the assembly of eIF4GI into tSGs and the rescue of tSG formation. **b**-**c** The expression of G3BP-ΔRBD (**b**) or 2A/L (**c**) interferes eIF4GI-G3BP interaction to block tSG assembly

Previous studies showed that L protein of encephalomyocarditis virus (EMCV), a cardiovirus that belongs to the *Picornaviridae* family, also inhibits AS-induced tSG formation but does not cleave eIF4GI^19^. To determine whether the inhibitory mechanism of L is similar to that of 2A, we expressed L^EMCV^ in HeLa cells and found that L^EMCV^ indeed blocked tSG formation (Fig. 7d, e) and disrupted G3BP-eIF4GI interaction (Fig. 7f, lane 5 vs 6). Taken together, our results show that 2A and L of picornaviruses block tSG formation by disrupting eIF4GI-G3BP interaction (Fig. 8).

## Discussion

Our previous study showed that 2A protease activity of picornaviruses is required for the blockage of tSG formation, but the molecular mechanism remains unknown. In this study, we found a general regulation mechanism of tSG formation: eIF4GI-G3BP interaction is critical for tSG formation under environmental stress, which was supported by five lines of evidence. First, we found that KD or KO of eIF4GI disrupted tSG formation (Fig. 1), suggesting that eIF4GI is critical for tSG formation. Second, although both TIA-1 and G3BP are RNA-binding proteins and important for tSG assembly, eIF4GI interacts with G3BP but not TIA-1 (Fig. 2c–e and Supplementary Fig. S2), suggesting that eIF4GI-G3BP interaction is specific. Third, aa 182–203 of eIF4GI are required for eIF4GI-G3BP interaction, and any variants or mutants lacking aa 182–203 could not interact with G3BP (Fig. 3b and d) or localize to tSGs (Fig. 3c, e and f). Even some eIF4GI variants such as eIF4GIΔ1-203 (known as eIF4GIa) were reported to form functional PICs^31,35^, suggesting that eIF4GI-G3BP interaction is required for stalled PIC assembly into tSGs in mammalian cells. Fourth, any eIF4GI mutants that no longer interacted with G3BP were unable to rescue tSG formation in HeLa-eIF4GI-KO cells when treated with AS (Fig. 4), suggesting that eIF4GI participates in SG formation via its association with G3BP. Of note, in the early stages of the construction of the monoclonal HeLa-eIF4GI-KO cell line, cells were unable to form tSGs, but as the culture time prolonged, the percentage of cells with tSGs gradually increased, and finally, about 40% of the cells were able to form tSGs when treated with AS. Because eIF4Gs are essential for cell survival, perhaps KO of eIF4GI caused the gradual enrichment of cells expressing more eIF4GII, which could partially compensate the function of eIF4GI in SG formation^36,37^. Fifth, the RBD of G3BP1 is required for eIF4GI-G3BP1 interaction, and the association of G3BP1-ΔRBD with G3BP1 disrupts eIF4GI-G3BP interaction (Fig. 5g and f), resulting in the inhibition of tSG formation (Fig. 5d, e). It suggested that G3BP1-ΔRBD binds to G3BP1 and changes the conformation of G3BP1, rendering heterozygous G3BP1-ΔRBD-G3BP complexes unable to bind to eIF4GI. These results were in accordance with previous study in which oligomerization of G3BP was required for AS-induced SG formation^3^. Therefore, we proposed a mechanism of SG formation–oligomer G3BP nucleates SGs via its interaction with eIF4G. The RGG region was previously reported to mediate the binding of G3BP1 to 40S ribosomal subunits^38^. Here, we found that G3BP1-ΔRGG still bound to eIF4GI (Fig. 5c), indicating that eIF4GI-G3BP interaction is independent of the association of G3BP with 40S ribosomal subunits. Furthermore, we also found that the eIF4GI-G3BP interaction is RNA-dependent. It is more likely that RNAs influence the space-conformation of G3BP, eIF4GI or both of them, which is required for eIF4GI-G3BP interaction and SG formation. SG assembly is driven by abundant of interactions of RNAs and proteins, it’s reasonable that RNAs transform the space-conformation of RNA-binding proteins to regulate SG assembly.

Next, we demonstrated that 2A or L protein of picornaviruses blocked tSG formation by disrupting eIF4GI-G3BP interaction. Of note, although aa 172–203 of eIF4GI were indispensable for eIF4GI-G3BP interaction (Fig. 3), eIF4GI-NT containing aa 172–203 failed to interact with G3BP (Fig. 6a). eIF4GI-CT also failed to interact with G3BP (Fig. 6a). Taken together, these data suggested that the full-length eIF4GI is required for eIF4GI-G3BP interaction, thus we hypothesize that the cleavage of eIF4GI by 2A may contribute to the blockage of eIF4GI-G3BP interaction. To our surprise, in cells expressing eIF4GI^G689E^, a cleavage-resistant mutation of eIF4GI, EV71 infection and the expression of 2A protease of picornaviruses still disrupted the eIF4GI^G689E^-G3BP interaction and inhibited tSG formation (Figs. 6–7); 2A^C110S^ could not block tSG formation and had no effect on eIF4GI^G689E^-G3BP interaction (Fig. 6e)^20^; in addition, L protein of EMCV could not cleave eIF4GI, but it still disrupted eIF4GI-G3BP interaction and blocked tSG formation (Fig. 7d, e), suggesting that, disruption of eIF4GI-G3BP interaction depends on 2A protease activity, but is not related to eIF4GI cleavage by 2A. Our results also prompt a new question: How does 2A/L protein disrupt eIF4GI-G3BP interaction? The various functions of 2A/L in picornavirus infection emphasize an unavoidable limitation in our study, whereby our results cannot distinguish between the direct and indirect inhibitory effects of 2A/L on eIF4GI-G3BP interaction. Further studies are needed to screen and characterize 2A/L-interacting proteins that may be involved in regulating eIF4GI-G3BP interaction.

Although previous studies showed that G3BP is a critical SG-nucleating factor^3,39–41^, its molecular mechanisms have not yet been fully understood. Furthermore, recent studies showed that SG formation/assembly involves three important steps--nucleation, growth, and fusion. Nucleation occurs when individual messenger ribonucleoproteins oligomerize into a core structure, which then grows via the recruitment of stalled PICs to form a more dynamic shell layer. Then, these PICs further fuse into a larger structure via the interactions of the shell layers to finally form a mature tSG^42–44^. Here, we found a new mechanism by which G3BP contributes to tSG formation via eIF4GI-G3BP interaction. We identified the specific point at which eIF4GI-G3BP interaction is involved in tSG formation. In cells expressing eIF4GI variants that could not interact with G3BP, AS-induced tSGs formed, but these eIF4GI variants could not assemble into tSGs, indicating that eIF4GI-G3BP interaction functions in recruitment of stalled PICs—i.e., the growth phase in SG assembly. Furthermore, 2A or L abolished tSG formation by blocking eIF4GI-G3BP interaction, suggesting that eIF4GI-G3BP interaction also functions in the early stage of SG assembly—nucleation. Unfortunately, we are currently unable to elucidate the mechanism of eIF4G-G3BP interaction-mediated tSG assembly in detail, but we hypothesize that eIF4G-G3BP interaction is the bridge between G3BP nucleation and stalled PICs recruitment. As a critical scaffold protein of PICs, eIF4GI connects the translation initiation factors, 40S ribosomes, and mRNA into functional PICs; thus, it is comprehensible that eIF4GI-G3BP interaction is much more efficient for nucleating and recruiting stalled PICs in the presence of stress. Therefore, eIF4GI-G3BP interaction might be the bridge between G3BP nucleation and stalled PICs recruitment, and provides a ubiquitous G3BP-mediated SG-nucleating and -growing mechanism.

Previous study showed that deletion of G3BP1/2 abolishes SGs triggered by p-eIF2α or eIF4A inhibition, but not those SGs induced by osmotic or heat stress^38^. Then, how does SG formation in the absence of G3BP in osmotic or heat stress? Some studies stated that the components of SGs induced by osmotic or heat stress were different from SGs triggered by p-eIF2α or eIF4A inhibition, such as heat-stress induced SGs containing heat shock proteins (HSPs)^45^. Exposure of cells to heat stress causes expression of a large amount of HSPs which prevent or reverse the inactivation of heat-sensitive proteins by interacting with HSPs^46^. Since the HSPs-interacting factors include many translation proteins, such as eIF4A and eEF1B^47^, we suspected that the function of HSPs interaction with translation proteins involved in tSG formation was similar as the eIF4G-G3BP interaction. For osmotic stress-induced SG formation, the function of eIF4G-G3BP interaction may be also supplemented by other osmotic stress-induced interactions.

In conclusion, our findings provide the first evidence that eIF4GI-G3BP interaction is indispensable for tSG formation. Furthermore, 2A or L protein of picornaviruses blocks eIF4GI-G3BP interaction, resulting in the blockage of tSG formation.

## Materials and methods

### Cell culture, infection, and transfection

HEK293T, rhabdomyosarcoma (RD) and HeLa cells were cultured in Dulbecco’s modified Eagle’s medium (DMEM) (Gibco) supplemented with 10% fetal bovine serum (FBS) (Gibco) and 100 U/ml penicillin/streptomycin (Gibco) at 37 °C and 5% CO_2_. The stably expressing (eIF4GI/eIF4GI^G689E^-HA-HeLa), KO (HeLa-eIF4GI-KO), or KD (sh-NC/sh-eIF4GI/sh-eIF4GII/sh-PABP/sh-NUP98/sh-NUP62/sh-FBP1/sh-SRF/sh-GAB1/sh-SQSTM1-HeLa) cells derived from HeLa cells were maintained in DMEM with 10% FBS, 100 U/ml penicillin/streptomycin, and 1 μg/ml puromycin (Sigma-Aldrich) at 37 °C and 5% CO_2_.

For infection, HeLa cells were infected with DMEM containing EV71 with a multiplicity of infection of 10 plaque-forming units. After 1 h incubation, the medium was replaced with fresh DMEM with 4% FBS, and this time point was considered 0 h post-infection.

For transfection, plasmids were transfected by using Lipofectamine 2000 (Invitrogen) according to the manufacturer’s instructions.

### SG induction and quantification

For SG induction, cells were treated with 100 μM AS (Sigma-Aldrich) for 1 h (or otherwise as indicated) before being harvested for further analysis.

For quantification of SGs, G3BP or TIA-1 was used as an SG marker. Cells were considered tSG positive only if they had SGs containing the indicated marker, and the diameter of the biggest SGs was at least 1.5 μm (the diameter of the biggest SGs in mock-treated cells was about 3–5 μm). For quantification of SGs in EV71-infected or 2A-expressing cells, Sam68 was a marker of EV71 infection or 2 A expression.

### Plasmids

The 2A proteinase of EV71-BrCr (NCBI accession no. U22521), PV (NCBI accession no. NC_002058.3), and CVA (NCBI accession no. KC117318.1), full-length G3BP (NCBI accession no. NM_005754.2), and eIF4GI (NCBI accession no. NM_001194947.1, isoform 6) were generated as described in our previous study^20^. The mutants of G3BP or eIF4GI were generated by standard molecular methods and cloned into the same vector as the wild-type. TIA-1 (NCBI accession no. NM_022037.2, isoform 1) was obtained from HeLa cells via RNA extraction and subsequent reverse transcription polymerase chain reaction and cloned into pWPI vector with an N-terminal Flag tag. The coding region of L protein of EMCV (NCBI accession no. X74312.1) was generated by chemosynthesis and cloned into the pCDNA3.0-IRES vector with a C-terminal Flag tag.

All of the structures were confirmed via DNA sequencing.

### Stable KD/overexpression cell lines

The stable KD/overexpression cell lines were generated as described in our previous study^20^. The target sequences for the shRNA constructs were: sh-NC, GCGCGATAGCGCTAATAATTT; sh-eIF4GI, GCCCTTGTAGTGACCTTAGAA; sh-eIF4GII, GCAGTTTCTGTAAACAACTTG; sh-PABP, CCAGACCTCATCCATTCCAAA; sh-NUP98, CCCTTGCAGATGGCTCTTAAT; sh-NUP62, TCTGGCACTGGAGGGTTTAAT; sh-FBP1, CGACCTGGTTATGAACATGTT; sh-SRF, CGATGTTTGCCATGAGTATTA; sh-GAB1, GCTGGATTGACATTTAACAAA; sh-SQSTM1, CCGAATCTACATTAAAGAGAA.

### eIF4GI KO cell lines

The eIF4GI KO cell line (HeLa-eIF4GI-KO) was constructed by using a CRISPR/Cas9 system and monoclonal screening. Briefly, gRNA (GGTGCAAGCCCTACAGAATTTGG) targeting the 7th exon of eIF4GI was synthesized and constructed into the pLentiCRISPR-v2 vector^48^. Then, lentiviruses were packaged in HEK-293T cells by co-transfection of the plasmids pLentiCRISPR-v2, psPAX2, and pMD2.G and then used to infect HeLa cells. The infected cells were then cultured under the selection pressure of 2 μg/ml puromycin to eliminate the non-gRNA-expressing cells. After the first round of screening, the cells were digested into single cells and seeded in 96-well plates with an average confluence of 0.8 cell/well. After culture, cells derived from monoclonal cells were amplified individually. Each cell line was validated via immunofluorescence (IF), Western blotting (WB), and sequencing. The targeting sequences were amplified via PCR using Taq polymerase and constructed into pMD18-T Simple Vector (Takara) using the following primer pair: Validate-F (TGGCTTGTCTTGTCTTGACCTA) and Validate-R (ATGAGTTTAGATGCTCCCTTGG). After validation, the HeLa- eIF4GI-KO cell line was used in subsequent experiments.

### Co-IP assays

Cells were harvested and lysed in 400 μl lysis buffer (150 nM NaCl, 50 nM Tris-HCl [pH 7.4], 1% Triton X-100, 1 mM EDTA [pH 8.0], and 0.1% sodium dodecyl sulfate [SDS]) with a protease inhibitor cocktail, incubated on ice for 30 min, and centrifuged for 30 min at 4 °C and 12,000×*g*. Then, one-tenth of the supernatants were boiled in SDS-polyacrylamide gel electrophoresis loading buffer at 100 °C for 10 min, and the lysates were analyzed via WB. The remaining supernatants were incubated on a spinning device with 20 ul anti-FLAG M2 affinity gel (Sigma-Aldrich) at 4 °C for 4 h. Beads were washed four times with 800 μl lysis buffer and then boiled in SDS sample buffer at 100 °C for 10 min to elute the immunoprecipitates. Alternatively, the remaining supernatants were incubated on a spinning device with 1 μg antibody or immunoglobulin G (IgG) previously conjugated to 50 μl protein G Dynabeads (Invitrogen) at 4 °C for 3 h. Beads were washed three times with 1X phosphate-buffered saline and then boiled in SDS sample buffer at 70 °C for 10 min to elute the immunoprecipitates, which were subjected to WB analysis. For RNase A-treated IP assays, the lysates were treated with 20 μg/ml RNaseA for 30 min at 16° C and then subjected to IP with anti-G3BP antibodies.

### IF and WB assays

For IF, cells were fixed and incubated with antibodies as described in our previous study^20^. The following dye-conjugated secondary antibodies were used for this analysis: Alexa Fluor 647 donkey anti-goat IgG H + L, Alexa Fluor 488 donkey anti-rabbit IgG H + L, and Alexa Fluor 594 donkey anti-mouse IgG H + L (Life Technologies). Cells were examined on a Leica confocal microscope.

For WB, samples were also analyzed as described in our previous study^20^ and detected on a Fujifilm LAS-4000 imaging system. The indicated primary and horseradish peroxidase-conjugated secondary antibodies (ThermoFisher Scientific) were used.

The primary antibodies goat polyclonal anti-TIA-1 (Cat#sc-1751) and mouse monoclonal anti-GAPDH (Cat#sc-32233) were purchased from Santa Cruz Biotechnology. Mouse monoclonal anti-G3BP (Cat#611127) was purchased from BD Transduction Laboratories. Rabbit polyclonal anti-eIF4E (Cat#A2162) was purchased from ABclonal. Rabbit monoclonal anti-eIF4GI (Cat#2858 S) and rabbit monoclonal anti-DYKDDDDK (Flag; Cat#14793 S) were purchased from Cell Signaling Technology. Mouse monoclonal anti-Flag (Cat#F1804), mouse monoclonal anti-HA (Cat#H9658), and rabbit monoclonal anti-HA (Cat#H6908) were purchased from Sigma-Aldrich. Mouse monoclonal anti-VP1 (Clone 22 A14) was purchased from Abmax^49^.

### Statistical analysis

Statistical analysis was performed using GraphPad Prism v6.01. All results are expressed as means ± standard deviation of at least three independent experiments (*n* ≥ 3). The *p* value was calculated using an unpaired Student’s *t*-test. In all tests, *p* > 0.05 was considered non-statistically significant (n.s.), and *p* < 0.05 was considered statistically significant, marked as follows: *p < 0.05; **p < 0.01; ***p < 0.001.

## Electronic supplementary material

Supplementary Information

## Electronic supplementary material

**Supplementary Information** accompanies the paper at (10.1038/s41421-018-0068-4).
