# Supplementary material for: SG formation relies on eIF4GI-G3BP interaction which is targeted by picornavirus stress antagonists
Source: Cell Discov. 2019 Jan 1;5:1. doi: 10.1038/s41421-018-0068-4 (PMC6312541; doi:10.1038/s41421-018-0068-4)
Supplement: Supplementary file 1 — Supplementary Information [file 41421_2018_68_MOESM1_ESM.pdf]

Supplementary Information

Supplementary Figures and Figure legends

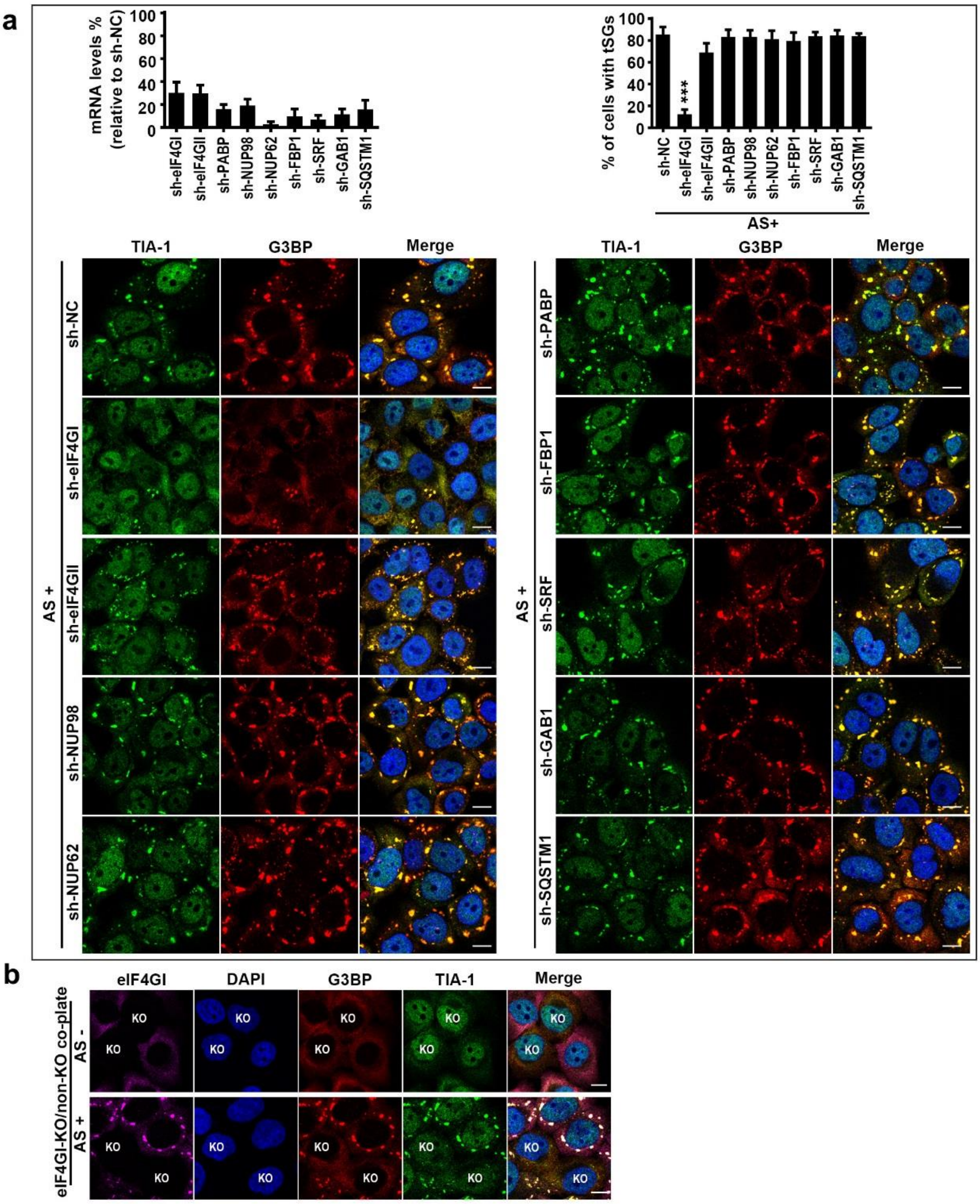

Figure S1. KD of substrates of 2A protease or KO of eIF4GI affects tSG formation.

a. Each 2A protease substrate was KD by shRNA in HeLa cells. The target mRNA

levels were measured by RT-q-PCR (top and left panel). Cells were treated with AS for 1 h and processed as in Figure 1b (bottom panel). Cells with tSGs were quantified as in Figure 1c (top and right panel).

b. HeLa (non-KO) and HeLa-eIF4GI-KO cells were co-plated and mock-treated or treated with AS for 1 h and then fixed and stained with eIF4GI (purple), G3BP (red), and TIA-1 (green).

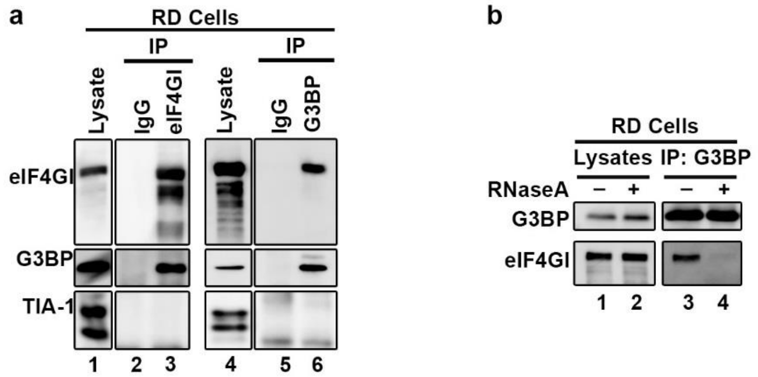

Figure S2. eIF4GI interacts with G3BP.

a. Interactions between endogenous eIF4GI and G3BP in RD cells were analyzed as in Figure 2d and 2e.

b. RD cells were lysed and treated with RNaseA (+) or mock-treated (-) before IP with anti-G3BP antibody, followed by detection of eIF4GI and G3BP via Western blots.

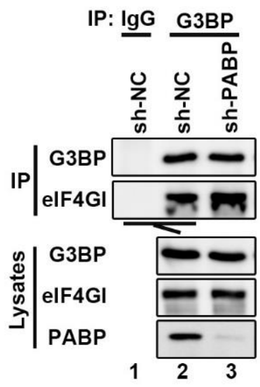

Figure S3. IP assays of G3BP binding with eIF4GI in HeLa cells with PABP depletion.

Sh-NC, and sh-PABP-HeLa cells were lysed and subjected to IP with anti-G3BP antibody, followed by Western blotting to resolve the indicated proteins.
